# Correction: SARS-CoV-2 suppresses IFNβ production mediated by NSP1, 5, 6, 15, ORF6 and ORF7b but does not suppress the effects of added interferon

**DOI:** 10.1371/journal.ppat.1010146

**Published:** 2021-12-10

**Authors:** Maya Shemesh, Turgut E. Aktepe, Joshua M. Deerain, Julie L. McAuley, Michelle D. Audsley, Cassandra T. David, Damian F. J. Purcell, Victoria Urin, Rune Hartmann, Gregory W. Moseley, Jason M. Mackenzie, Gideon Schreiber, Daniel Harari

S4 Fig is a duplicate of S3 Fig. Please view the correct S4 Fig below.

## Supporting information

S4 Fig IFNα and IFNγ transcription levels are not altered by MAVS or SARS-CoV-2 genes. HEK-293T cells were transfected with MAVS and a SARS-CoV-2 viral gene (or control). 24 hours post transfection transcript levels were analyzed by qPCR for expression of IFNα2, IFNα4, IFNα6, IFNα10 and IFNγ. The data presented are expression levels normalized to the housekeeping gene HPRT1 (ΔCT). Data presented are means of 2–4 independent experiments and their standard error.
(TIF)
