# Supplementary material for: SARS-CoV-2 suppresses IFNβ production mediated by NSP1, 5, 6, 15, ORF6 and ORF7b but does not suppress the effects of added interferon
Source: PLoS Pathog. 2021 Aug 26;17(8):e1009800. doi: 10.1371/journal.ppat.1009800 (PMC8389490; doi:10.1371/journal.ppat.1009800)
Supplement: S1 Fig — (A) Graphical map of SARS-CoV-2 (NCBI) shows the relative positioning of SARS-CoV-2 genes. The polygenic ORF1a and ORF1b (ORF1ab) transcripts encode genes NSP1-NSP11 and NSP1-NSP16, respectively. (B) RNAseq data from SARS-CoV-2 infected Calu3 or VERO E6 cells (published by Finkel el. al., Nature 589:125, 2021) using normalized RPKM (Reads Per Kilobase Million) compared to qPCR data from HEK-293T cells transfected with individual SARS-CoV-2 expressing genes. The transfected viral genes included a downstream IRES and puromycin gene. Thus qPCR measurements for each viral gene was performed indirectly detecting puromycin. Small genes (ORF6 and ORF7b) were not added to the RNAseq data due to small transcript size and lack of robust read numbers. In both RNAseq and qPCR studies, gene expression was expressed as fold-change ratio in relation to the housekeeping gene, HPRT1. (PDF) [file ppat.1009800.s001.pdf]

A

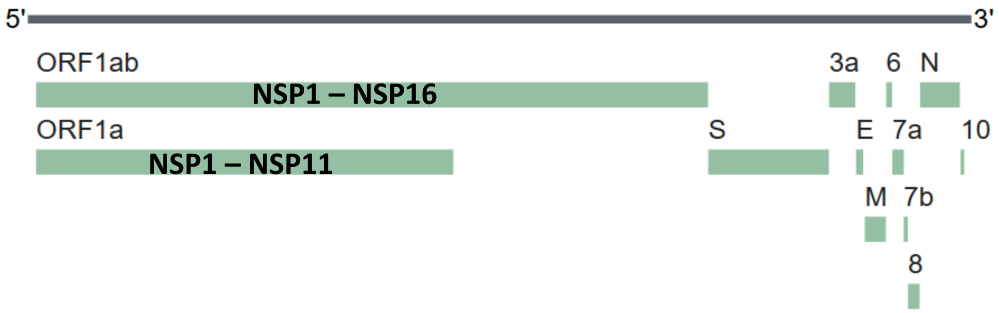

B

Gene Expression relative to HPRT1 Control

Gene	CALU (7 Hrs)	VERO (5Hrs)	SEGMENT	HEK-293T
ORF1a	23.2	31.5		
ORF1b	38.6	34.3		
NSP1-NSP11	61.8	65.8	NSP6	20.6
			NSP8	36.3
NSP13-NSP15	38.6	34.3	NSP13	31.1
			NSP14	14.3
			NSP15	14.8
S	108.8	49.3		1.4
ORF3a	178.5	86.6		49.9
ORF3b				60.0
E	222.5	21.4		35.2
ORF6				10.6
ORF7B				28.2
ORF8	324.0	151.4		30.1
M	753.0	319.1		68.8
N	1936.4	906.3		92.5
GFP	Not Relevant	Not Relevant		224.6
HPRT1	1.0	1.0		1.00
