# Supplementary figures and images for: SARS-CoV-2 suppresses IFNβ production mediated by NSP1, 5, 6, 15, ORF6 and ORF7b but does not suppress the effects of added interferon
Source: PLoS Pathog. 2021 Aug 26;17(8):e1009800. doi: 10.1371/journal.ppat.1009800 (PMC8389490; doi:10.1371/journal.ppat.1009800)

A

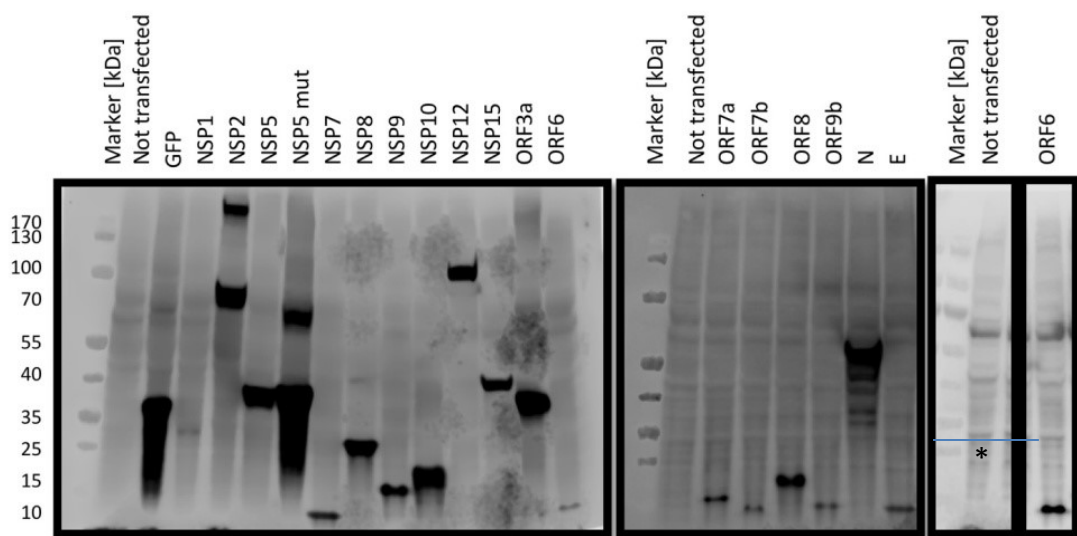

B

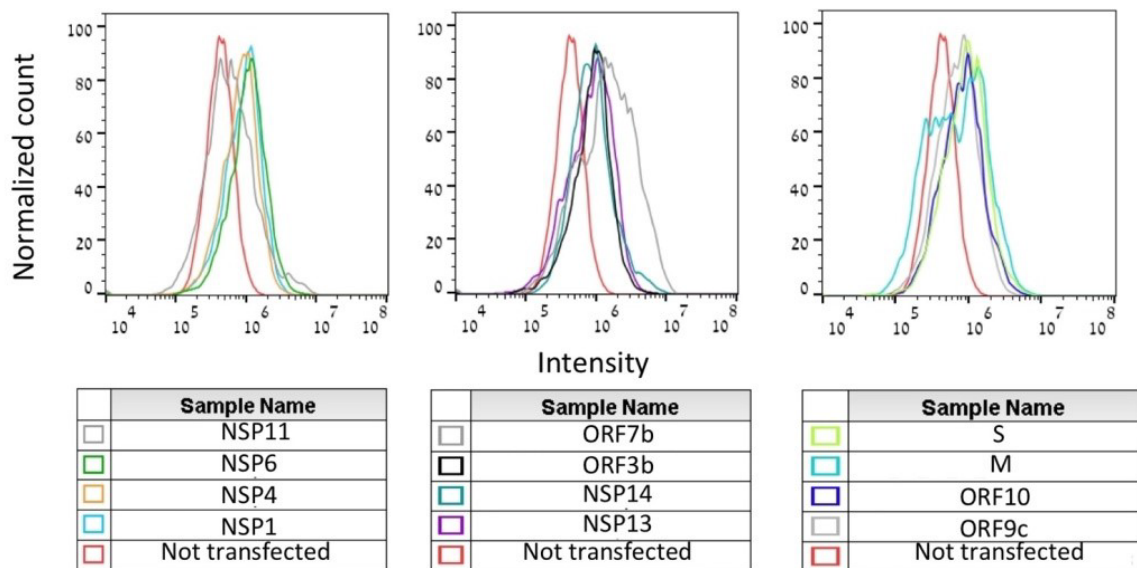

C

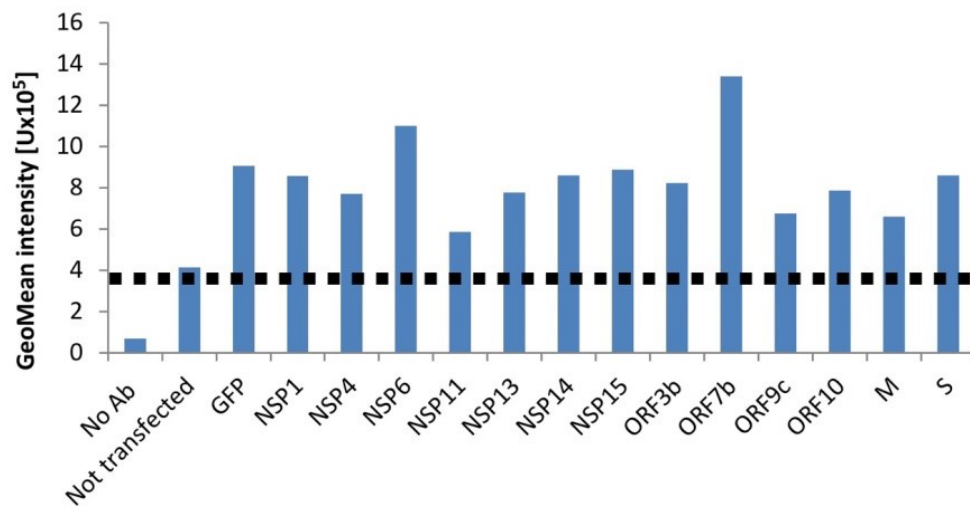

Supplement: S2 Fig — Verification of viral protein expression—HEK-293T cells were transfected with vectors encoding viral proteins fused to a 2xStrep tag. 24 hours post transfection cells were collected and analyzed by western blot (A) or FACS (B, C), using specific strep tag antibody. Samples were compared to the not-transfected control. (C) Quantitative analysis of the GeoMean intensity obtained in (B). The dashed line indicates background signal, as determined by the non-transfected control. *: The two last panels on the right-hand-side in (A) are from the same membrane but with irrelevant intervening samples removed. (PDF) [file ppat.1009800.s002.pdf]

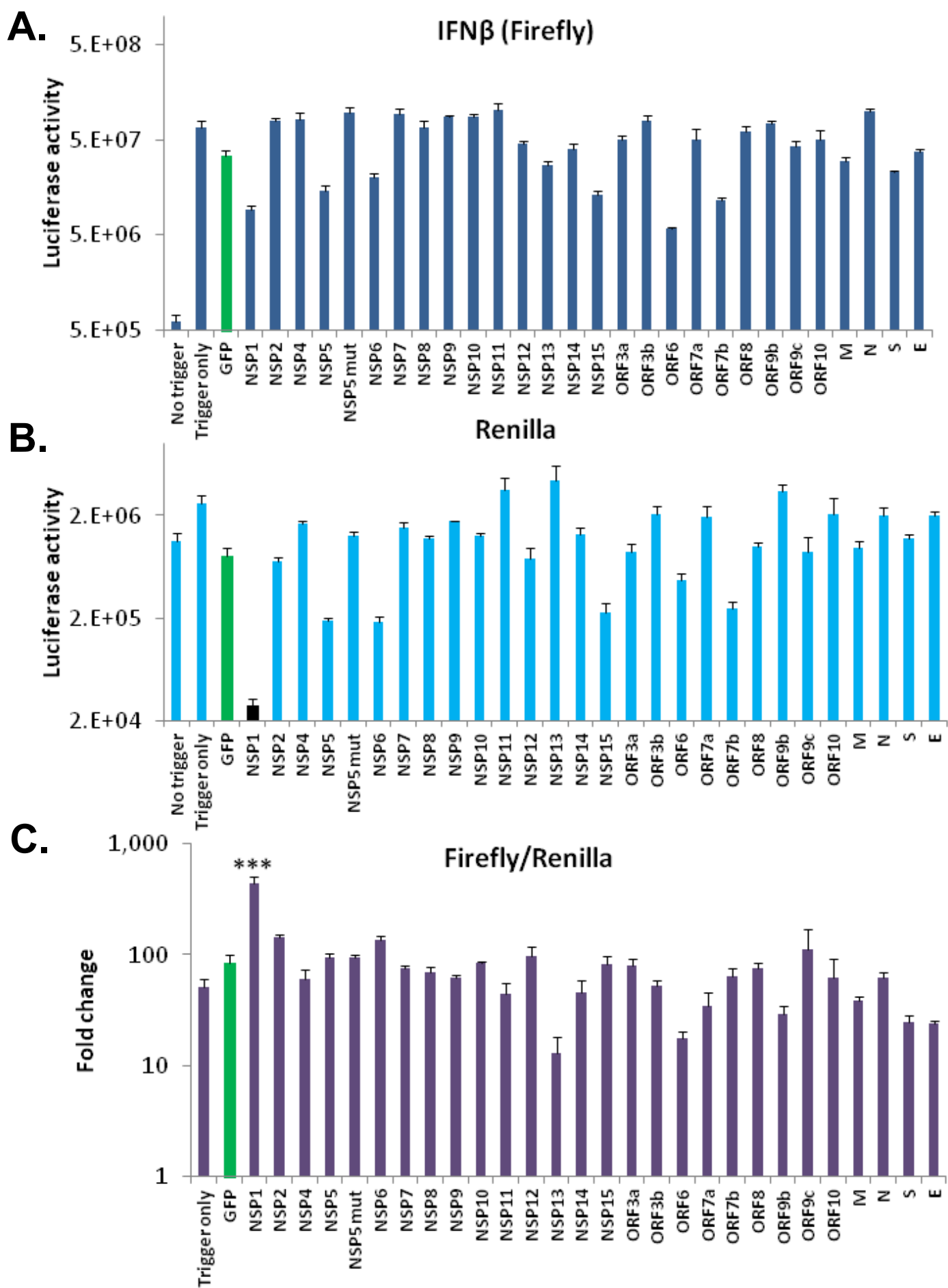

Supplement: S3 Fig — HEK-293T cells were transfected with IFNβ promotor-Firefly luciferase, Renilla luciferase, TRIF and a SARS-CoV-2 viral gene (or control). ’No trigger’ refers to cells transfected with Firefly and Renilla only; ’Trigger only’ refers to cells transfected with all components but the viral gene. 24 hours post transfection luciferase activity was measured. Firefly (A) and Renilla (B) activities are presented separately. Normalized activity of Firefly/Renilla, also normalized with basal activity (no trigger), is presented in (C). Data presented are means calculated based on four independent experiments and their standard error. (PDF) [file ppat.1009800.s003.pdf]

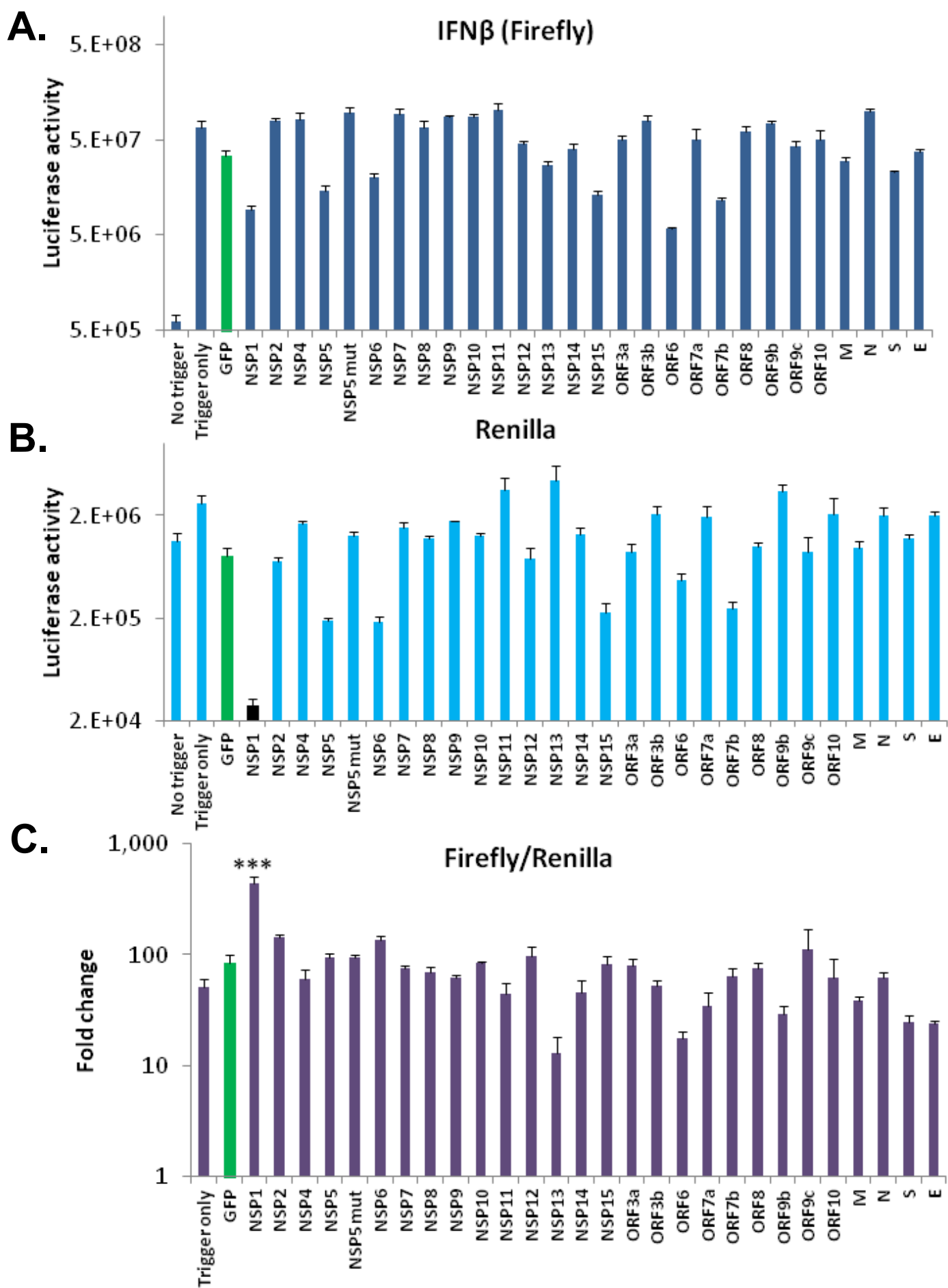

Supplement: S4 Fig — HEK-293T cells were transfected with MAVS and a SARS-CoV-2 viral gene (or control). 24 hours post transfection transcript levels were analyzed by qPCR for expression of IFNα2, IFNα4, IFNα6, IFNα10 and IFNγ. The data presented are expression levels normalized to the housekeeping gene HPRT1 (ΔCT). Data presented are means of 2–4 independent experiments and their standard error. (PDF) [file ppat.1009800.s004.pdf]

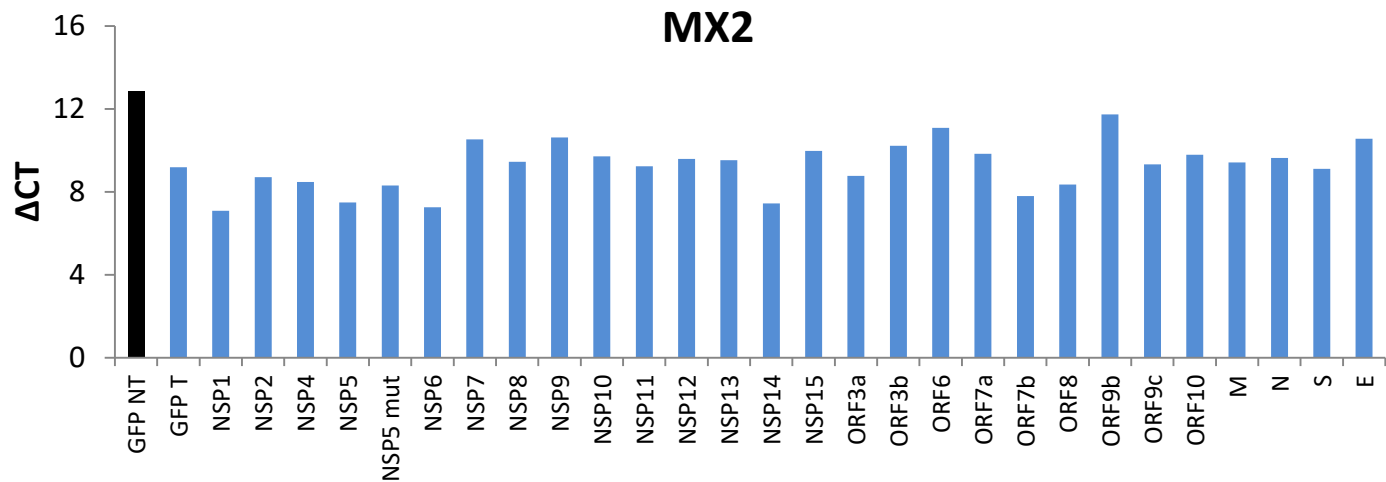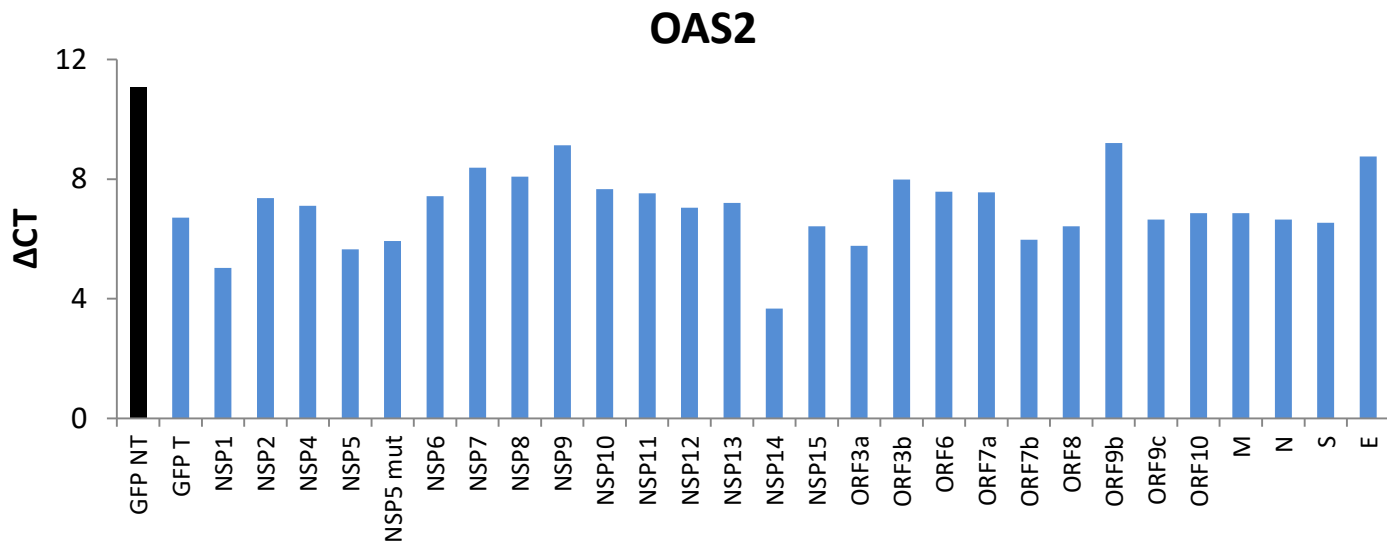

Supplement: S5 Fig — HEK-293T cells were transfected with a viral gene. 24 hours post transfection cells were treated with low concentration (10 pM) IFNβ for additional 24 hours. Transcript levels were analyzed by qPCR, and normalized with the housekeeping gene HPRT1 (ΔCT). The GFP control was either treated (T) or not treated (NT; marked in black) with IFNβ. (PDF) [file ppat.1009800.s005.pdf]

A

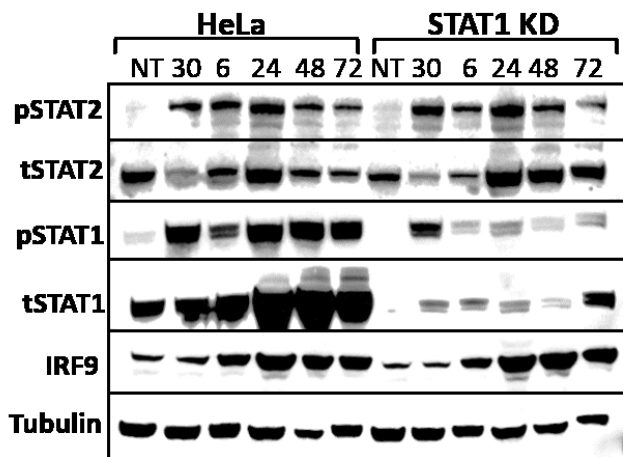

B

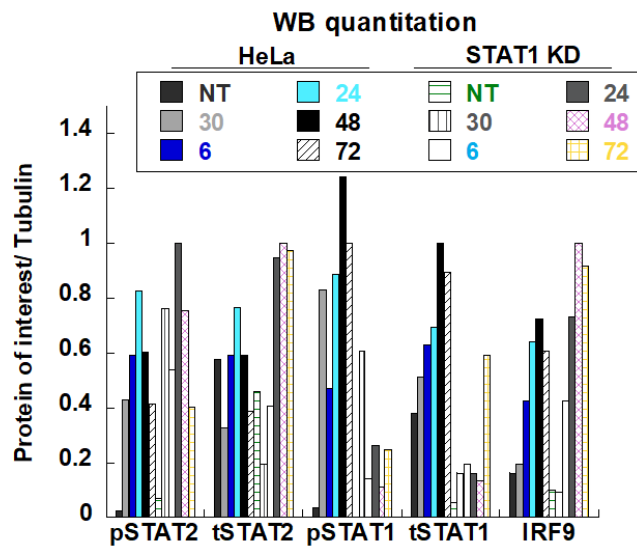

C

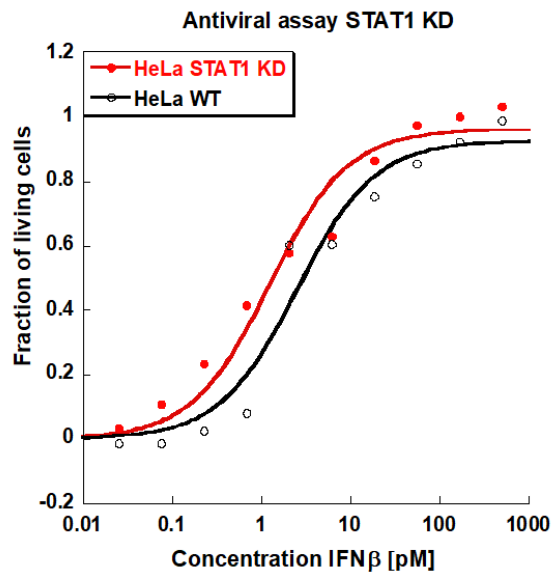

D

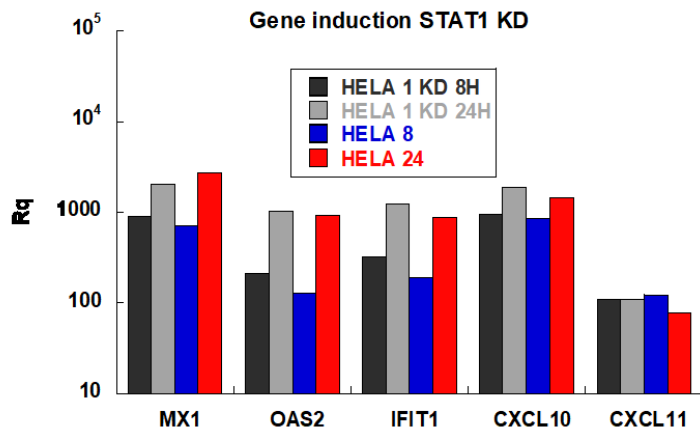

Supplement: S6 Fig — (A) STAT1 KD and control HeLa cells were treated with 2nM IFNβ for the indicated time points and STAT protein levels were assessed by western blot. (B) Quantification of (A). (C) STAT1 KD and control HeLa cells were treated with serial dilutions of IFNβ (starting with 500pM). 4 hours later, cells were infected with Vesicular Stomatitis Virus (VSV) and after a further 18 hours, the plate was stained for cell density using crystal violet. (D) STAT1 KD and control HeLa cells were treated with 2nM IFNβ for 8 or 24 hours and then analyzed by qPCR. The data presented show the relative fold-change expression levels in relation to untreated cells after normalization with HPRT1. NT: Not treated. (PDF) [file ppat.1009800.s006.pdf]
