# Supplementary material for: SARS-CoV-2 suppresses IFNβ production mediated by NSP1, 5, 6, 15, ORF6 and ORF7b but does not suppress the effects of added interferon
Source: PLoS Pathog. 2021 Aug 26;17(8):e1009800. doi: 10.1371/journal.ppat.1009800 (PMC8389490; doi:10.1371/journal.ppat.1009800)

A. Transfection efficiency for IFN transcript level analysis

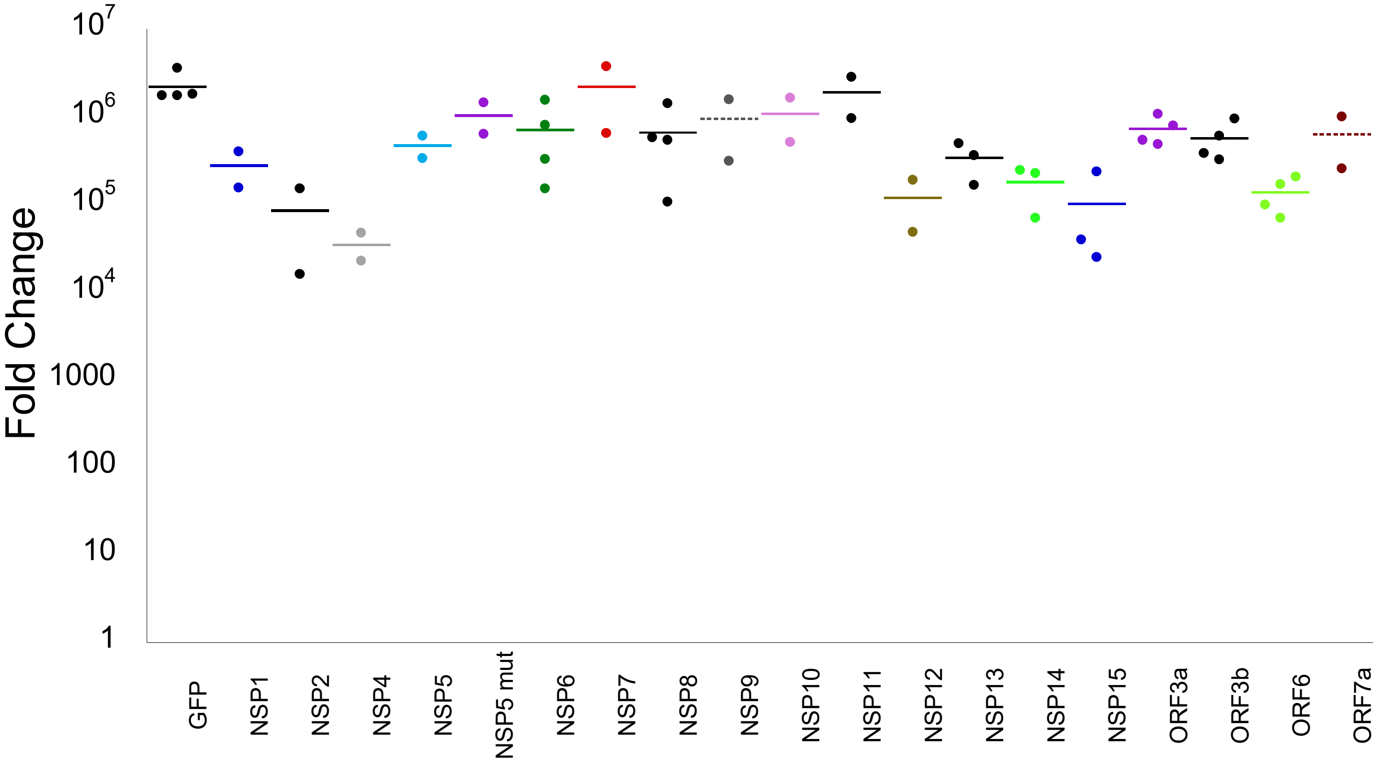

B. Transfection efficiency for ISGs transcript level analysis

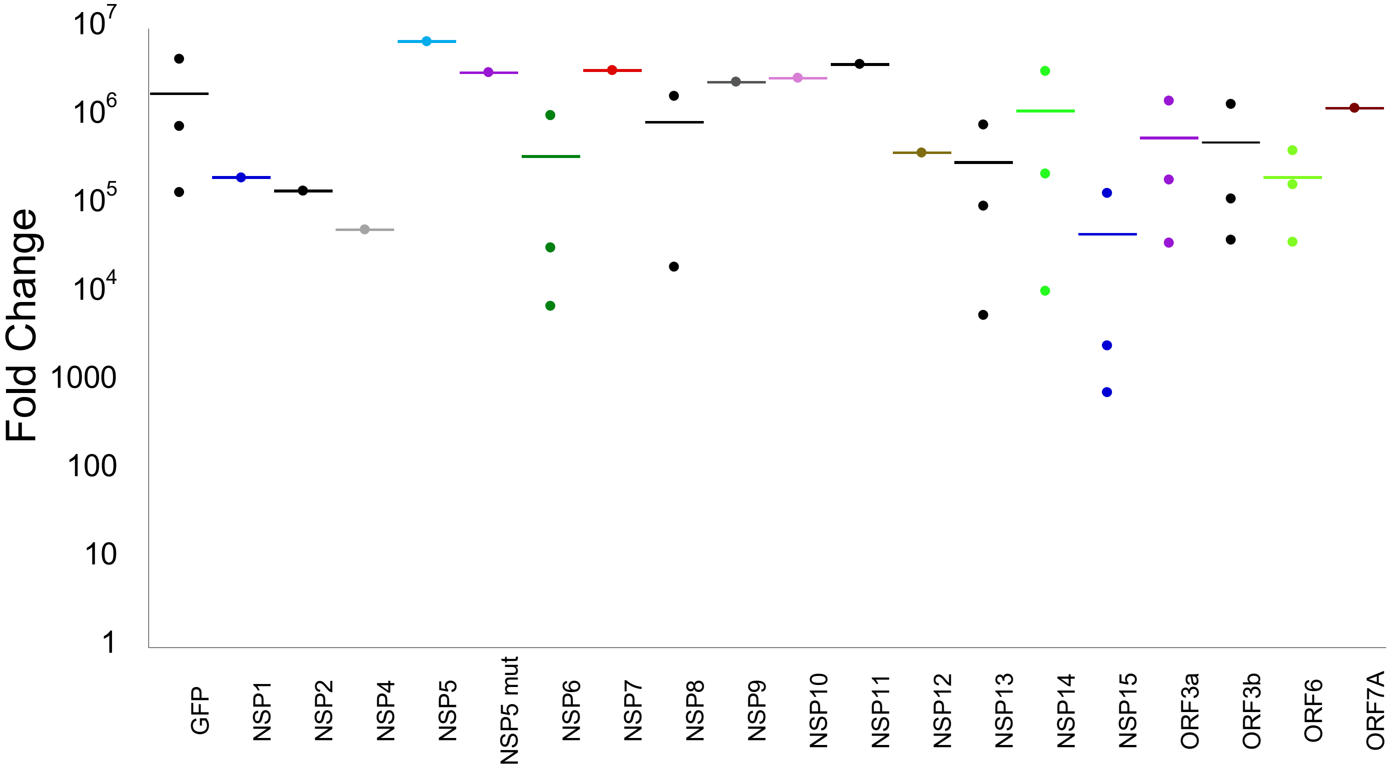

Supplement: S7 Fig — (A) HEK-293T cells were transfected with MAVS and a viral gene. 24 hours post transfection transcript levels were analyzed by qPCR (see Fig 4). Note: Some of data used to derive S1B Fig overlaps with data used to generate the graph shown here. (B) HEK- 293T cells were transfected with a viral gene. 24 hours post transfection cells were treated with 100 pM IFNβ for additional 24 hours and analyzed by qPCR (see Fig 6). All viral genes harbors a common downstream bicistronic puromycin resistance gene, which serves to evaluate transfection efficiency by qPCR. The data presented here are puromycin expression levels normalized with the housekeeping gene HPRT1 (ΔCT) of the individual repeats. (PDF) [file ppat.1009800.s007.pdf]
