# Supplementary material for: SARS-CoV-2 suppresses IFNβ production mediated by NSP1, 5, 6, 15, ORF6 and ORF7b but does not suppress the effects of added interferon
Source: PLoS Pathog. 2021 Aug 26;17(8):e1009800. doi: 10.1371/journal.ppat.1009800 (PMC8389490; doi:10.1371/journal.ppat.1009800)
Supplement: Attachment — Submitted filename: Response to reviewers_DH.docx [file ppat.1009800.s009.docx]

## **Date: 29^th^ June, 2021**

## **Subject: Response to Reviewers**

**Article Title:**	SARS-CoV-2 suppresses IFNβ production mediated by NSP1, 5, 6, 15, ORF6 and ORF7b but does not suppress the effects of added interferon.

**Authors:**	Maya Shemesh **^1,^** ^¶^, Turgut E. Aktepe **^3,^** ^¶^, Joshua M. Deerain **^3^**, Julie L. McAuley **^3^**, Michelle D. Audsley **^2^**, Cassandra T. David **^2^**, Damian F. J. Purcell **^3^**, Victoria Urin **^1^**, Rune Hartmann **^4^**, Gregory W. Moseley **^2^**, Jason M. Mackenzie **^3^**, Gideon Schreiber **^1,*^** and Daniel Harari

Dear Editor and reviewers,

We thank you for your positive feedback and recommendations for improvement of this manuscript. We have used this time to perform additional experiments in response to your requests. This has been incorporated into a new manuscript, which we co-submit along with our collaborators together with this letter. With exception of our editing minor grammatical/spacing/figure number changes in the new resubmitted document, for your convenience we have highlighted in yellow all other changes made.

Below, please find our response to your comments and recommendations.

Thanking you for your time and your useful comments in reviewing our paper, and hoping you find our new modifications to your liking.

Sincerely yours,

Daniel Harari

--------------------------------------------------------------------------------------------------------------------

**Editor:** Both reviewers found the study interesting, important and thorough. Both had some good suggestions that will strengthen the conclusions that can be drawn from the work.

Reviewer's Responses to Questions

**Part I - Summary**
Please use this section to discuss strengths/weaknesses of study, novelty/significance, general execution and scholarship.

Reviewer #1: (No Response)

Reviewer #2: ‘SARS-CoV-2 suppresses IFNβ production 1 mediated by NSP1, 5, 6, 15, ORF6 and ORF7b but does not suppress the effects of added interferon’ by Maya Shemesh et al provides a detailed examination of 27 SARS-CoV-2 genes on interferon production. The authors examine MAVS and TRIF mediated IFNb signaling pathways with all genes and also examine a subset of genes and their ability to inhibit ISG expression. Six SARS-CoV-2 genes were found to block IFNb signaling although nsp1 was shown to have a global effect on expression. The manuscript is very thorough and the authors have taken care to consider previous work in this area and put their results into the context of previous studies. The summary in table 1 is very helpful. Overall, this is an extensive and detailed analysis but the heavy reliance on transfection and RNA data and number of previous publications in this area are serious weaknesses.

**Part II – Major Issues: Key Experiments Required for Acceptance**
Please use this section to detail the key new experiments or modifications of existing experiments that should be absolutely required to validate study conclusions.

Generally, there should be no more than 3 such required experiments or major modifications for a "Major Revision" recommendation. If more than 3 experiments are necessary to validate the study conclusions, then you are encouraged to recommend "Reject".

Reviewer #1: (No Response)

Reviewer #2: Expression data for transfected genes as presented is odd/incomplete. It is far preferable to show protein by western blot.

**RESPONSE:** Your point is pertinent. We have added a new section to the text in which we have utilized detection of recombinant viral proteins by immunological detection of a 2xSTEP tag found in all of SARS-CoV-2 gene constructs. Here we have demonstrated now protein expression by either Western Blot or by FACS (Figs. S2A and S2B). We will keep the transcriptional data analysis as well (Fig S1) because this approach allows for comparison of transcript expression between transfected genes and that of SARS-CoV-2 infected cells, this which cannot be done by immunological analysis for lack of appropriate antibody reagents.

Lines (304-309) and new supplementary figure (Fig. S2):

“*In addition to transcript levels we verified viral protein translation, exploiting detection of the proteins via their fused 2xStrep tag [42]. Translation was determined using western blot (Fig. S2A) or FACS (Fig. S2B and C). All of the SARS-CoV-2 proteins were expressed, albeit not necessarily correlating with that for transcript levels (Fig. S1 and also Fig. S7A and B). In summary, all recombinant viral genes were demonstrated to be expressed at both the transcript and protein levels.”*

We have also appended the relevant protocol additions to the Materials and Methods section.

Figure 5 – It is important to consider that the cumulative action of several SAR2 proteins could suppress ISG expression when a single gene product cannot.
**RESPONSE**: We agree with this point. It is for this very reason that this assay was repeated using SARS-CoV-2 infected cells (was old Fig. 7F 🡪 Now new Fig. 8F), where we show no change in the level of gene expression for four ISGs upon viral infection (with expression of a viral gene being used as a positive control to demonstrate infection).

Figure 5 – needs a positive control showing that they can block ISG induction from this level of IFNb treatment, it is possible that the system is being overwhelmed.

**RESPONSE**: In the results section (lines 426-430) we have added:

*“To account for saturation of the system by this concentration of IFNβ (100 pM), which may mask an inhibitory effect on the ISGs by viral genes, we repeated this experiment with 10 pM IFNβ (Fig. S5). We observed here weak expression of MX2 and OAS2 with IFNβ treatment. Once again, none of the transfected SARS-CoV-2 genes blocked expression of these ISGs (Fig. S5), supporting that SARS-CoV-2 does not directly act to suppress ISG production.”*

*.*

Regarding the reviewer’s request that we add a positive control to the assay, we attempted this with the Mumps-V protein, this which causes a reduction of cellular STAT1 levels. Using this “positive control” we failed to block ISG activation. This is consistent with the findings for SARS-CoV-2 NSP1 in our own study. A clue which is likely to explain this apparent contradiction in results is shown Fig. S6: Knockdown of STAT1 in Hela cells resulted in a sharp reduction of STAT1 levels. This correlated nicely with a block in pSTAT activation (Fig S6 A&B) but nevertheless these cells also retained robust IFN-induced antiviral response (Fig. S6 C) and IFN-induced ISG activation (Fig. S6 D). We conclude that only small amounts of the total pool of STAT1 are required to elicit a full IFN-induced anti-viral response.

**Part III – Minor Issues: Editorial and Data Presentation Modifications**
Please use this section for editorial suggestions as well as relatively minor modifications of existing data that would enhance clarity.

Reviewer #1: I enjoyed reading this paper. The data are valuable for understanding not only SARS-CoV-2 biology but also potential therapeutic approaches. The experiments are rigorous and the conclusions are justified by the data presented. I have some minor comments:

The authors indirectly show a decrease in virus-infected cells after addition of IFNa by qPCR. It would be worth repeating this with more direct evidence of decrease in virus.
Since the authors show that ifnb is upregulated by MAVS, this assay should be re-done with both IFNA and IFNB.

**RESPONSE**: We have done as the reviewer has requested. We deemed the finding of this study important enough to create a new figure for the manuscript (new Fig. 1) because it provides a biological context for the rest of the manuscript. Here we tested directly the anti-viral effects of both IFNα and IFNβ in SARS-CoV-2 infected Vero cells, This is shown in (lines 268-285):

***“Type I IFN treatment diminishes infectious virus titer of SARS-CoV-2***

*We first tested if added IFN-Is have the capacity to diminish SARS-CoV-2 infection. To do this, Vero cells were infected at a MOI of 0.01 and after 6 hours were treated with 500 IU/mL of either IFNα or IFNβ and incubated for an additional 18 hours before determining viral titer by TCID50 assay. Both type I IFN variants induced a sharp reduction (>10-fold) in viral titer (Fig. 1). These results demonstrate that, added IFN-Is have the capacity to diminish the advance of SARS-CoV-2 infection. This is an important and fundamental finding regarding the biology of SARS-CoV-2 in response to added interferons and may have therapeutic implications. However, this assay does not provide crucial insight into the dynamics of the natural IFN-I response in an infected host. Namely, 1). Does early stage SARS-CoV-2 viral infection trigger the natural production of IFN-Is in a host? and 2). The observed decrease in viral titer to added IFN may be because of direct anti-viral effects taking place within an infected host cell. Alternatively (or additionally) added IFNs may be triggering the anti-viral activity in uninfected cells adjacent to the infected cell, thus perturbing advance of viral spread. For the remainder of this study we focus upon determining if/which SARS-CoV-2 genes have the capacity to inhibit IFN-I production. We also examined if/which of these viral genes have the capacity to block the innate antiviral response. These questions were addressed studying IFN-activation and response in both virus-infected cells as well as by studying individual SARS-CoV-2 genes transfected to mammalian cells.”*

*”*

Accompanying changes have been made in the Figure section and Materials and Methods (lines 257-271)*.*

Also in the Abstract we have added (lines 26-27): *“In sharp contrast, SARS-CoV-2 infected cells remained extremely sensitive to anti-viral activity exerted by added IFN-Is.”*

Reviewer #2: Figure 1 – The black and green bars coloration is explained in the figure legend, should also indicate what blue vs gray means in the legend.

**RESPONSE**: We have now added to the figure legend (for a new Figure 2):

*“Grey bars in histograms: Minor (< 10-fold) repression of MAVS-induced IFNβ activity.”*

Figure 3 legend – should refer to parts A, B and C

**RESPONSE:** Done (for what is now Figure 4)

Line 339 – “do not directly assess the amount of IFNb protein…” – these assays don’t measure protein at all. Please use more precise phrasing.

**RESPONSE:** We have removed the word “directly” (Lines 401-402).

Why not do an ELISA to measure IFNb protein levels?

**RESPONSE:** Using TCA to precipitate IFNβ from conditioned medium has the advantage that it allowed us to concentrate the secreted cytokine into a small volume, thus increasing the possibility that we could detect the naturally secreted protein, with western blot assuring the identity of the protein. We performed quality control analysis by testing if/how different amounts of purified IFNβ added to a gel will demonstrate signal after SDS-PAGE/WB. The results are given below here:

In line 209-213 of Materials and Methods section we have added:

*“This experimental system was validated by running SDS-PAGE samples harboring increasing concentrations of purified IFNβ (50, 100, 200 & 400 nM; each in 30ul volume) prior to WB analysis. Here we observed a band of increasing intensity at the expected mobility of ~22KDa. Densitometry analysis furthermore demonstrated a linear relationship between IFN concentration and Unit intensity.”*

The linear relation between the loaded quantity of IFNβ and signal density clearly shows that the relative levels of production of IFNβ upon SARS-CoV-2 gene transfection, as provided in Fig. 5B are relevant. ELISA would be an alternative method, but may be less sensitive as the protein would have been much more diluted.

Line 360-361 – Should say that 4 ISGs were measured?

**RESPONSE:** Fixed (now lines 421-422)

Why the switch from IFNb to IFNa stimulation between figures 5 and 6?

**RESPONSE:** Experiments were performed across different institutions using different reagents. The Australian labs (Peter Doeherty and Monash) primarily used IFNα and the Israeli lab used IFNβ. The point raised here was addressed indirectly by the other reviewer of this paper where he/she requested that we repeat direct measurements of SARS-CoV-2 infection using both of these cytokines, this which we have now performed. We show in Fig. 1 that administration of both IFNs result in similar reduction in virus titer.

Figure 7 labels – what is RbStat1? **RESPONSE:** This has been changed to Anti-STAT1 and anti-pSTAT1 (Now Figure 8)

Figure 9 legend – please mention the star present on the nsp1 images **RESPONSE:** (Now Figure 10) In the legend it is now written: *“Stars * - NSP1-transfected cells (green) demonstrate a sharp reduction in puromycin signal (red).”*

Line 571 – “SARS-CoV-2 does not suppress IFN-activation” – should this read ISG activation?

**RESPONSE:** Correct. This statement has been removed. The last paragraph of the discussion (lines 646-658) has been changed, and includes the correction of this incorrect statement.

**Additional Change:**

In light of the new data in our study (particularly Fig. 1), we have changed the final paragraph in the manuscript, trying to link between our findings and that of possible clinical application (lines 631-647):

*“In conclusion, our work supports a multi-gene viral-mediated blockade of MAVS-induced IFN production corresponding to that of early stages SARS-CoV-2 infection of lung cells. This blockade in IFN production provides the virus with an evolutionary advantage to remain in “stealth mode”, in order to delay immune detection. Of practical application, our findings thus support that IFN-Is can be useful to treat early-stage SARS-CoV-2 infection. Additionally, IFN-treatment could have prophylactic application for identified individuals exposed to SARS-CoV-2 patients, where the chain of disease transmission can be curtailed or perhaps even stopped. It is not at all clear however if IFN therapy has clinical application for patients with well-established infection, where the host IFNαs are endogenously being produced. A possible exception to this counter argument however, is that for a large subset (~10%) of patients with life-threatening COVID-19, neutralizing antibodies to IFN-Is (particularly IFNα and IFNω) have been detected in patient serum, this which is almost never found in the general population, and of which is almost certainly blocking the potent anti-viral effects of IFN-Is, hence contributing to the severe pathology [78]. For this subset of patients with life-threatening COVID-19, we suggest that therapeutic intervention with IFNβ may have life-saving effects, particularly because added IFNβ provides a robust anti-viral response to SARS-CoV-2 cells (Fig. 1) and due to its divergent sequence to other IFN-Is, should escape IFNα/IFNω antibody-mediated neutralization.”*

We thank you for your most useful comments and for your review of our manuscript.

Regards,

Daniel Harari & co-authors.
