# Supplementary figures and images for: Correction: SARS-CoV-2 suppresses IFNβ production mediated by NSP1, 5, 6, 15, ORF6 and ORF7b but does not suppress the effects of added interferon
Source: PLoS Pathog. 2021 Dec 10;17(12):e1010146. doi: 10.1371/journal.ppat.1010146 (PMC8664222; doi:10.1371/journal.ppat.1010146)

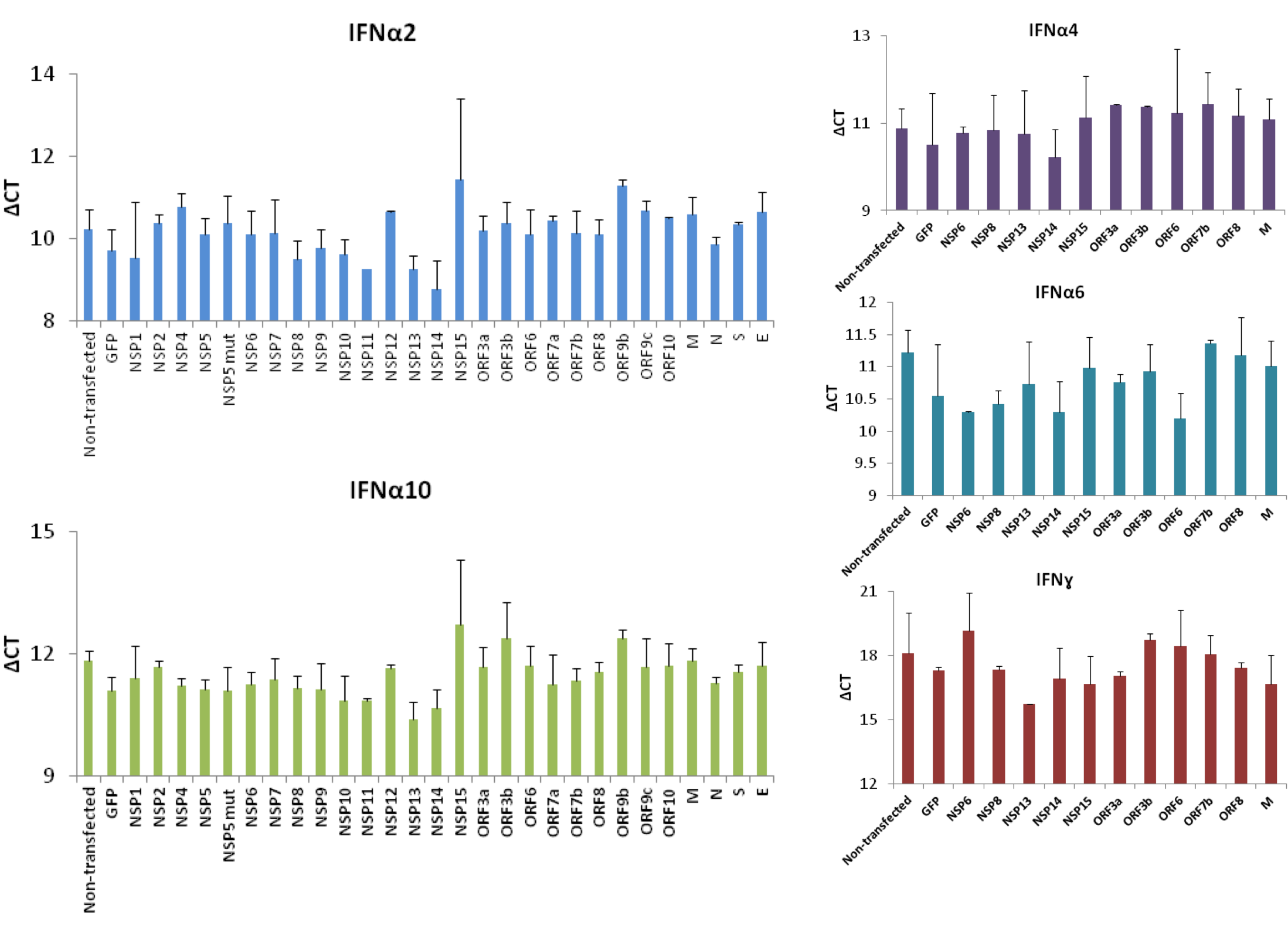

Supplement: S4 Fig — HEK-293T cells were transfected with MAVS and a SARS-CoV-2 viral gene (or control). 24 hours post transfection transcript levels were analyzed by qPCR for expression of IFNα2, IFNα4, IFNα6, IFNα10 and IFNγ. The data presented are expression levels normalized to the housekeeping gene HPRT1 (ΔCT). Data presented are means of 2–4 independent experiments and their standard error. (TIF) [file ppat.1010146.s001.tif]
